# High prevalence of viral hepatitis in a series of splenic marginal zone lymphomas from Romania

**DOI:** 10.1038/bcj.2016.102

**Published:** 2016-11-11

**Authors:** B Fetica, B Pop, M L Blaga, A Fulop, D Dima, M T Zdrenghea, C I Vlad, A S Bojan, P Achimas-Cadariu, C I Lisencu, A Irimie, D D Weisenburger

**Affiliations:** 1 Department of Pathology, The Oncology Institute ‘Prof. dr. I. Chiricuta', Cluj-Napoca, Romania; 2 Department of Pathology, University of Medicine and Pharmacy ‘Iuliu Hatieganu', Cluj-Napoca, Romania; 3 Department of Epidemiology and Biostatistics, The Oncology Institute ‘Prof. dr. I. Chiricuta', Cluj-Napoca, Romania; 4 Department of Hematology, The Oncology Institute ‘Prof. dr. I. Chiricuta', Cluj-Napoca, Romania; 5 Department of Hematology, University of Medicine and Pharmacy ‘Iuliu Hatieganu', Cluj-Napoca, Romania; 6 Department of Surgery, The Oncology Institute ‘Prof. dr. I. Chiricuta', Cluj-Napoca, Romania; 7 Department of Surgery and Gynecologic Oncology, University of Medicine and Pharmacy ‘Iuliu Hatieganu', Cluj-Napoca, Romania; 8 Department of Pathology, City of Hope National Medical Center, Duarte, CA, USA

Marginal zone lymphoma is a heterogeneous category of indolent non-Hodgkin lymphomas (NHLs). Splenic marginal zone lymphoma (SMZL) represent 2% of lymphoid neoplasia.^1^ Epidemiologic studies have linked this category of NHLs to several agents capable of establishing chronic infections in humans.^2^ Hepatitis C virus (HCV) infection has been linked especially with SMZL.^2, 3^

In this paper, we present 34 retrospectively selected cases of SMZL representing 4.65% of all lymphomas and 6.0% of all NHLs in a series of 731 consecutive lymphoma cases diagnosed between 1 January 2006 and 29 February 2016, in the Department of Pathology at the Institute of Oncology 'Prof. Dr. Ion Chiricuta', Cluj-Napoca, Romania. Data collection was performed in March and April of 2016. The number of SMZL cases was double compared with the expected incidence reported for this disease.^1^ Of the 17 cases with available status for viral hepatitis and human immunodeficiency virus (HIV) infection 5 cases were diagnosed with hepatitis B (HBV) or C infection (29%).

All tissue samples were fixed in 10% neutral buffered formalin. For bone marrow biopsy (BMB) specimens, decalcification was performed in ethylenediaminetetraacetic acid with disodium salt acid buffer (Osteodec, Bio-Optica, Milan, Italy) for a duration of 3 h, followed by paraffin embedding. Sections were cut at 4 μm and stained using hematoxylin and eosin, and Gomori for the evaluation of fibrosis.

Immunohistochemistry was performed by using the following antibodies: CD20 (mouse monoclonal antibodyL26, ABCAM (Cambridge, UK), dilution 1:50), CD3 (polyclonal rabbit anti-human antibody, clone UCHT1, Dako, Glostrup, Denmark; dilution 1:100), CD45 (mouse monoclonal antibody clone 2B11, Dako, dilution 1:100), CD10 (mouse monoclonal antibody, clone 56C6, Novocastra, Buffalo Grove, IL, USA; dilution 1:100), CD5 (mouse monoclonal antibody clone 4C7, Novocastra, dilution 1:100), CD23 (mouse monoclonal antibody, clone 1B12, Novocastra, dilution 1:50), BCL2 (mouse monoclonal antibody, clone 124, Dako, dilution 1:100), BCL6 (mouse monoclonal antibody, clone BL6.02, Thermo Scientific, Waltham, MA, USA; dilution 1:20), cyclin D1 (mouse monoclonal antibody, clone DCS-6, Santa Cruz, dilution 1:100), CD79a (mouse monoclonal antibody, clone JCB117, Dako, dilution:1:50), Ki-67 (mouse monoclonal antibody, clone MM1, dilution 1:200), CD138 (monoclonal mouse antibody, clone MI15, Dako, dilution 1:25), CD43 (mouse monoclonal antibody, clone DF-T1, Dako, dilution 1:100), CD21 (mouse monoclonal antibody, clone 2G9, Novocastra, dilution 1:20), CD35 (mouse monoclonal antibody, clone E11, ABCAM, dilution 1:50) and CD11c (mouse monoclonal antibody, clone 5D11, Novocastra, dilution 1:100). Pre-treatment of tissues with heat-induced epitope retrieval was performed. The visualizations step employed the Novolink Polymer Detection System, Novocastra. Examination of all cases was performed by a pathologist with expertize in hematopathology (BF).

Screening for viral infections included the detection of the HBsAg, AgHBe antigens and anti-HBe, anti-HCV and anti-HIV 1 and 2 antibodies performed using the electrochemiluminescent immunoassay (ECLIA, Roche Diagnostics, Rotkreuz, Switzerland). Screening for hepatitis D (DELTA) included the detection of anti-HD antibodies and was performed using an enzyme-linked immunosorbent assay (ELISA). Viral load determination was performed using the real-time polymerase chain reaction(RT-PCR; Roche Diagnostics).

Of the 731 lymphoma cases, Hodgkin lymphoma represented 160 cases (21.9%) and the other 571 cases were diagnosed as NHLs (78.1%). From this series, 34 cases were diagnosed as SMZL, representing 4.7% of all lymphoma cases and 6.0% of all NHLs.

The SMZL cases had a male-to-female ratio of 1.8 (22 males and 12 females). The age of the patients ranged from 39 to 77 years with a median of 63 years. There was no significant difference between the median ages of males versus females (60 versus 66 years, K-sample equality-of-medians test *P*>0.05). The results of tests for viral hepatitis and HIV infection were available for 17 cases. Overall, five cases were diagnosed with hepatitis B or C infection (29.4%). Of these, two were anti-HCV positive (11.7%) and three (17.7%) were HBsAg positive (Table 1). All cases were HIV and HDV (Delta) negative. RT-PCR for viral load was available for one case with HCV infection (11833459 UI/ml) and one case with HBV infection (352218 UI/ml). One out of three HBV cases showed production of anti-HBe antibodies.

BMB was performed on all 17 patients (Table 1). In addition, splenectomy specimens were examined from two patients, and lymph node biopsy specimens were examined from two patients. BMB showed involvement by lymphoma in all cases with a predominantly interstitial pattern of infiltration (12/17 cases, 70.6%), followed by an intrasinusoidal pattern (9/17 cases, 53%). Paratrabecular, nodular and diffuse patterns of infiltration were less represented (Table 1). Reactive T-cell infiltrates were also observed in six cases (35.3%). In three cases, focal mild fibrosis was observed. The lymphoma infiltrate consisted of atypical small lymphocytes or medium-sized lymphocytes with moderate amounts of clear cytoplasm. Two cases showed transformation to diffuse large B-cell lymphoma (DLBCL) confirmed by positive lymph node biopsies. Of the two cases with DLBCL transformation, one was HCV positive and the other was HBV positive. One case developed a recurrence in a salivary gland.

Immunohistochemically, the lymphoma cells were positive for pan-B-cell markers (CD20, CD79a) and for common leukocyte antigen (CD45), and were negative for CD5, CD23 (one case had focal weak positivity), cyclin D1, CD3, CD10, CD11c (one case was positive) and CD30. Bcl2 was positive in two out of the three cases tested. One case was CD43 positive (on a recurrence in the parotid gland). Reactive T-cell infiltrates were CD3 and CD5 positive. Spleen involvement was observed in the white and red pulp with a predominantly nodular pattern.

The median follow-up of the 34 SMZL cases was 51 months. Of the 17 cases with available viral infection status, 12 (70.6%) were alive at the time of analysis. Three patients died in the HBV/HCV+ group and two in the HBV/HCV– group. A comparison of survival showed a borderline statistical difference between the two groups (log-rank *P*=0.055, Figure 1).

Herein, we report on a cohort of 34 patients with SMZL taken from a consecutive series of 731 lymphoma cases diagnosed in a single institution in Romania, over a period of 10 years, which represented 4.65% of all lymphoma cases and 6.0% of all NHLs. This percentage is twice as high as that reported in the literature.^1^ However, we suspect an even higher frequency of SMZL in our region given the fact that the disease is often underdiagnosed, and given the high incidence of HBV and HCV infection in our region (see below). The morphologic and immunohistochemical findings in our series (Table 1) are similar to that described in the literature.^1^ Survival analysis showed a borderline statistical difference between the HBV/HCV+ and HBV/HCV– cases (Figure 1), but this finding should be viewed cautiously given the small number of cases and the variable therapies used. Interestingly, transformation to DLBCL was observed only in the HBV/HCV+ group.

The frequency of HCV infection in our cases of SMZL was 11.7%, whereas the prevalence of the HCV infection in our region is 3.2–4.6%.^4, 5, 6^ HCV genotype 1 is the most common subtype (>50% of cases) in Romania.^5^ Several studies have established a strong link between the HCV infection and the development of SMZL including observations of complete or partial remissions of SMZL after antiviral therapy.^1, 2^ The frequency of HCV infection in SMZL patients is quite variable, ranging from 4.0 to 22%.^1, 3, 7, 8^

We also report a high frequency of HBsAg positive SMZL cases (17.7%) in our series. Due to public health campaigns the incidence of HBV infection, in Romania decresed from 43 cases per 100 000 persons in 1989^(ref.9)^ to 2.4 in 2010.^4, 6^ The prevalence of HBV infection, in Romania, is estimated at 5.6%.^4^ The relationship between SMZL and HBV infection has not been extensively studied in the literature. Koot *et al.*^10^ have reported a complete remission of SMZL after the control of the HBV viral load with tenofovir. Christou *et al.*^11^ and Mathew *et al.*^12^ have also reported two cases of SMZL in patients with HBV infection, and Zhang *et al.*^13^ and Iannitto *et al.*^14^ reported two HBV positive patients who developed SMZL and hepatocellular carcinoma. Gómez-de la Fuente *et al.*^15^ also reported a case of SMZL with reactivation of a past HBV infection. However, we were not able to identify reports of the prevalence of HBV infection in patients with SMZL.

As the relationship between HBV and NHL is still a matter of debate, establishing a connection between SMZL and HBV will most likely be challenging due to the rarity of SMZL and the variable incidence of HBV infection worldwide. We believe that the study of this relationship in areas of high HBV prevalence will have a greater chance of success. A solution for the ascertainment of cases of all cases of SMZL in our region would be the establishment of a lymphoma registry in the region.

In conclusion, we report a high frequency of HBV- and HCV-positive SMZL lymphoma cases in an area with a high prevalence of HBV and HCV infection. Given the increasing evidence of involvement of HBV and HCV in the development of NHL, the relationship between these viruses and SMZL should be carefully investigated in future studies.

## Figures and Tables

**Figure 1:**
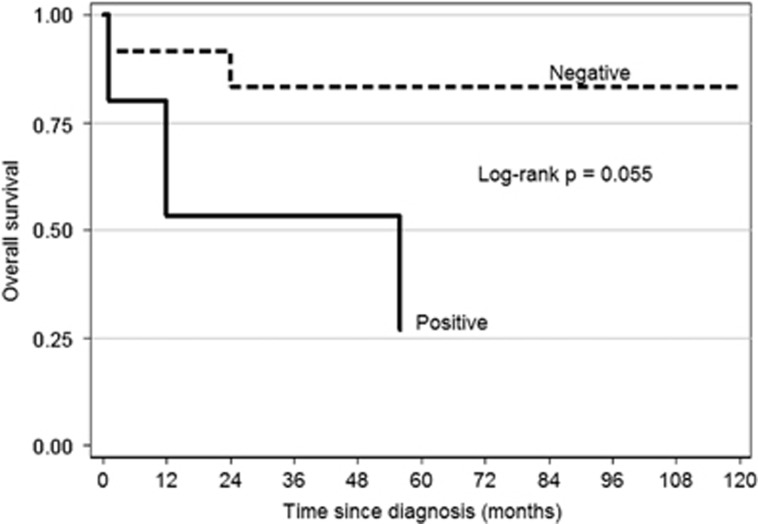
Overall survival estimates for 17 SMZL patients by viral hepatitis B or C status.

**Table 1:** Detailed presentation of the 17 SMZL patients with known viral infection status

*Case*	*Morphology (specimen, infiltration pattern)*	*Phenotype*	*Viral hepatitis status*	*Therapy*	*Observations*
1	BMB- interstitial, paratrabecular and intrasinusoidal with minimal fibrosis	CD20 + CD23, CD5, CD11c –.	HBV positive	CHOP Splenectomy R-ICE Partial response Lamivudine	Recurrence one year later
2	BMB- interstitial, intrasinusoidal Spleen- white and red pulp, diffuse infiltration by small lymphocytes with abundant cytoplasm and rare immunoblasts Lymph node (splenic hilum)- interfollicular, with focal follicular colonization Lymph node (retroperitoneal)- diffuse infiltrate of medium to large cells with 60% proliferation index(Ki-67)	CD20+ CD5, CD3, CD43, CD10, cyclin D1–(BMB). CD20+ CD5, CD3, CD43, CD10, cyclin D1–(spleen, splenic lymph node). CD20+ CD5, CD3, CD10–,(retroperitoneal lymph node)	HBV positive	COP Splenectomy R-CHOP R-FC Partial response Lamivudine	Transformation to DLBCL 3.4 years after initial diagnosis
3	BMB- interstitial, intrasinusoidal, with minimal fibrosis	CD20+ CD3, CD5; CD23–.	HBV positive	Active monitoring	
4	BMB- interstitial	CD20+ CD3, CD5, CD23–.	HCV positive	Not available	
5	BMB - interstitial, intrasinusoidal, nodular Axillary lymph node – DLBCL	CD45, CD20, CD79a+ BCL2, cyclin D1, CD56–.	HCV positive	CHOP with a complete response	Transformation to DLBCL 3 months after initial diagnosis
6	BMB- interstitial, intrasinusoidal	CD20, BCL-2 + CD5, CD23-;	Negative	R-CHOP Splenectomy	Stable disease with persistent lymphocytosis
7	BMB- diffuse	CD20+ CD23 focal+ CD5, CD10, cyclin D1,-;	Negative	Not available	
8	BMB- paratrabecular	CD20, BCL2+ CD10, CD3, CD30–.	Negative	VCAEP CHOP Partial response	Persistent IgM monoclonal gammopathy after chemotherapy
9	BMB- interstitial, intrasinusoidal,	CD20+ CD5, CD23, CD3–.	Negative	Splenectomy Complete response	
10	BMB- diffuse	CD20+ CD5, CD43, CD23, CD11C, cyclin D1–.	Negative	R-FC Partial good response	
11	BMB- nodular, interstitial, intrasinusoidal	CD20+ CD5, CD43, CD23, cyclin D1, CD11c–.	Negative	Chlorambucil R-COP Partial response	
12	BMB- interstitial, intrasinusoidal	CD20, CD45+ CD5, CD23, CD3–.	Negative	No therapy Stable disease	
13	BMB- interstitial, intrasinusoidal Parotid gland - nodular and diffuse infiltrates	CD20+ CD23, CD5, CD3–(BMB). CD20, bcl2, CD5, CD43, IgD+ CD35 weak and focal+ CD10, BCL6, cyclin D1–(parotid gland).	Negative	R-CHOP Splenectomy R-COP Partial response	Recurrence in parotid gland 7 years after initial presentation Persistent IgM monoclonal gammopathy after therapy
14	BMB- interstitial, with minimal, focal, fibrosis	CD20, CD11c+ CD5, CD3–.	Negative	Cladribrin Stable disease	
15	BMB- diffuse Spleen - white and red pulp involvement	CD20, CD 79, CD 43+, CD5 weak focal +, cyclin D1, CD3,CD11c–(BMB); CD20+, CD5, cyclin D1, CD3, CD11c, CD23–(spleen).	Negative	Splenectomy Partial response	Persistent lymphocytosis
16	BMB - interstitial	CD20+ CD3, CD5, CD23, cyclin D1–	Negative	FLUCYD R -COP Complete response	
17	BMB - paratrabecular	CD20+ CD3, CD5, CD23, cyclin D1–	Negative	Splenectomy No other data available.	

Abbreviations: BMB, bone marrow biopsy; COP, cyclophosphamide, oncovin (vincristine), prednisone; CHOP, cyclophosphamide, hydroxydaunorubicin, oncovin (vincristine), prednisone; DLBCL, diffuse large B-cell lymphoma; FluCyD, fludarabine, cyclophosphamide, dexamethasone; HBV, hepatitis B; HCV, hepatitis C; IgD, Immunoglobulin D; IgM, Immunoglobulin M; R-CHOP, rituximab+cyclophosphamide, hydroxydaunorubicin, oncovin (vincristine), prednisone; R-COP, rituximab+cyclophosphamide oncovin (vincristine) prednisone; R-FC, rituximab+ fludarabine cyclophosphamide; R-ICE, rituximab ifosfamide carboplatin etoposide; VCAEP, vincristine, cyclophosphamide, adriblastine, etoposide, prednisone.
